# Poly[diaqua­tetra-μ-seleno­cyanato-cadmium(II)dipotassium(I)]

**DOI:** 10.1107/S1600536810034938

**Published:** 2010-09-08

**Authors:** Thorben Reinert, Jan Boeckmann, Inke Jess, Christian Näther

**Affiliations:** a Institut für Anorganische Chemie, Christian-Albrechts-Universität Kiel, Max-Eyth-Strasse 2, 24098 Kiel, Germany

## Abstract

In the title compound, [CdK_2_(NCSe)_4_(H_2_O)_2_]_*n*_, the cadmium(II) cation is situated on a twofold rotation axis and is coordinated in a slightly distorted tetra­hedral geometry by two symmetry-related μ-1,1,1,3 and two-symmetry related μ-1,1,3,3 bridging seleno­cyanate anions, all of which are Se bonded. These bridging seleno­cyanate anions are further coordinated to two symmetry-related potassium ions. Each of the potassium ions is coordinated by one terminally bonded water mol­ecule and six seleno­cyanate anions, two of which are crystallographically independent. The asymmetric unit consists of one cadmium and one potassium cation, two bridging seleno­cyanate anions and one water mol­ecule. The polymeric subunits are further connected *via* the seleno­cyanate anions into a three-dimensional coordination network. In this coordination network, intramolecular hydrogen bonds between neighbouring water molecules can be found.

## Related literature

For general background to transition metal thio- and seleno­cyanates and *N*-donor ligands, see: Näther *et al.* (2007 ▶); Bhosekar *et al.* (2006 ▶); Wriedt & Näther (2010 ▶); Wriedt *et al.* (2010*a* ▶,*b* ▶). For related structures, see: Shi *et al.* (2007 ▶); Couhorn & Dronskowski (2004 ▶). For similar coordination modes in azido anions, see: El Fallah *et al.* (2008 ▶); Guo & Mak (1998 ▶).
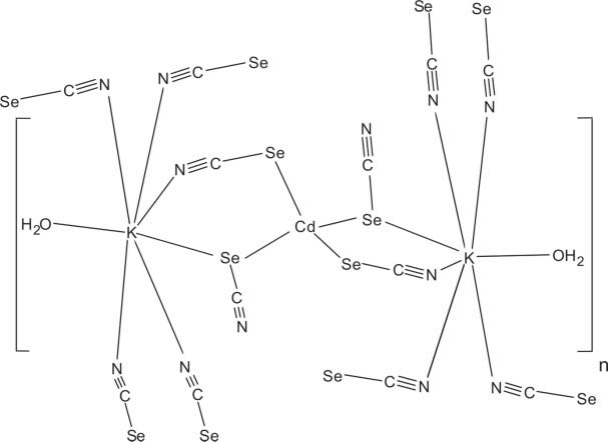


## Experimental

### 

#### Crystal data

[CdK_2_(NCSe)_4_(H_2_O)_2_]
*M* *_r_* = 646.55
Monoclinic, 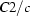

*a* = 21.574 (3) Å
*b* = 4.4055 (4) Å
*c* = 17.9316 (19) Å
β = 112.454 (13)°
*V* = 1575.1 (3) Å^3^
*Z* = 4
Mo *K*α radiation
μ = 11.15 mm^−1^
*T* = 170 K
0.05 × 0.04 × 0.03 mm

#### Data collection

Stoe IPDS-1 diffractometer
Absorption correction: numerical (*X-SHAPE* and *X-RED32*; Stoe & Cie, 2008 ▶) *T*_min_ = 0.588, *T*_max_ = 0.713
4687 measured reflections
1800 independent reflections
1355 reflections with *I* > 2σ(*I*)
*R*_int_ = 0.057

#### Refinement

*R*[*F* ^2^ > 2σ(*F* ^2^)] = 0.037
*wR*(*F* ^2^) = 0.090
*S* = 1.00
1800 reflections
79 parameters
H-atom parameters constrained
Δρ_max_ = 0.90 e Å^−3^
Δρ_min_ = −0.94 e Å^−3^

### 

Data collection: *X-AREA* (Stoe & Cie, 2008 ▶); cell refinement: *X-AREA*; data reduction: *X-AREA*; program(s) used to solve structure: *SHELXS97* (Sheldrick, 2008 ▶); program(s) used to refine structure: *SHELXL97* (Sheldrick, 2008 ▶); molecular graphics: *XP* in *SHELXTL* (Sheldrick, 2008 ▶); software used to prepare material for publication: *SHELXL97*.

## Supplementary Material

Crystal structure: contains datablocks I, global. DOI: 10.1107/S1600536810034938/fj2330sup1.cif

Structure factors: contains datablocks I. DOI: 10.1107/S1600536810034938/fj2330Isup2.hkl

Additional supplementary materials:  crystallographic information; 3D view; checkCIF report

## Figures and Tables

**Table 1:** Hydrogen-bond geometry (Å, °)

*D*—H⋯*A*	*D*—H	H⋯*A*	*D*⋯*A*	*D*—H⋯*A*
O1—H2⋯O1^i^	0.85	1.92	2.760 (6)	167

Symmetry code: (i) 
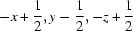
.

**Fig. 1.**
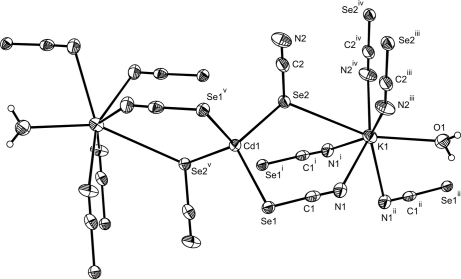
: Crystal structure of the title compound with labelling and displacement ellipsoids drawn at the 50% probability level. Symmetry codes: i: x, y + 1, z; ii: -x + 1/2, -y + 3/2, -z + 1; iii: -x + 1, -y + 1, -z + 1; iv: -x + 1, -y + 2, -z + 1; v: -x + 1, y, -z + 3/2; vi: x, y - 1, z.

**Fig. 2.**
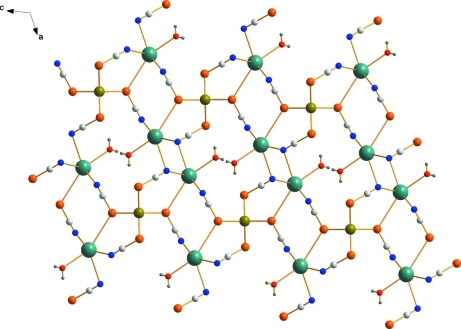
: Crystal structure of the title compound with view along the crystallographic b-axis.

## supplementary crystallographic information

### Comment

In our ongoing investigations on the synthesis, structures and properties of transition metal thio- and selenocyanates and N-donor ligands (Näther, Bhosekar & Jess (2007); Bhosekar *et al.* (2006); Wriedt & Näther (2010); Wriedt, Jess &  Näther (2010*a*,*b*)) we have reacted cadmium(II) dinitrate with potassium selenocyanate and pyrimidine in water in order to prepare cadmium selenocyanato coordination polymers with pyrimidine as co-ligand. In this reaction single crystals were obtained, which were identified as the title compound by single-crystal X-ray diffraction.

In the title compound of composition [CdK_2_(NCSe)_4_(H_2_O)_2_]*_n_* (Fig. 1) the cadmium cation is located on a twofold rotation axis and is coordinated to ten potassium cations *via* two µ-1,1,1,3 bridging and two µ-1,1,3,3 bridging selenocyanato anions. The cadmium cation is coordinated by Se atoms of four selenocyanato anions in a slightly distorted tetrahedral geometry. The Cd—Se distances range between 2.655 (7) Å and 2.672 (8) Å and the Se—Cd—Se angles range between 106.68 (21)° and 116.96 (17)° (Tab. 1). The potassium cations are each heptacoordinated by three N-atoms of three µ-1,1,1,3 bridging selenocyanato anions, two N-atoms of two µ-1,1,3,3 bridging selenocyanato anions, one Se-atom of one µ-1,1,3,3 bridging selenocyanato anion and one terminally bonded water molecule within an irregular geometry. The K—N distances range between 2.805 (69) Å and 3.083 (71) Å, the K—O distance amounts to 2.763 (45) Å and the K—Se distance is 3.694 (16) Å. The angles around the K atoms range between 63.10 (2)° and 155.51 (19)° (Tab. 1). The large K—Se and K—K distances are not unusual and similar values can be found in related structures (Shi *et al.*, 2007; Couhorn & Dronskowski, 2004). The Cd and K atoms are connected by the bridging selenocyanato anions into a three-dimensional coordination network (Fig. 2). It must be noted that the present bridging modes of the selenocyanato anions are observed for the first time in a coordination polymer and similar coordination modes can be found for azido anions (El Fallah *et al.*, 2008; Guo & Mak, 1998).

### Experimental

Cd(NO_3_)_2_*x* 4H_2_O was obtained from Merck, KNCSe and pyrimidine were obtained from Alfa Aesar. 1 mmol (174 mg) Cd(NO_3_)_2_*x* 4H_2_O, 2 mmol (288 mg) KNCSe, 4 mmol (320 mg) pyrimidine and 3 ml acetonitrile were reacted in a closed snap-cap vial without stirring. After the mixture had been standing for several days in the dark at room temperature colourless block shaped single crystals of the title compound were obtained.

### Refinement

The O—H hydrogen atoms were located in difference map, set to idealized distances and refined isotropic using a riding model with *U*_eq_(H) = 1.5**U*_eq_(O).

### Crystal data

**Table:**

[CdK_2_(NCSe)_4_(H_2_O)_2_]	*F*(000) = 1176
*M**_r_* = 646.55	*D*_x_ = 2.726 Mg m^−^^3^
Monoclinic, *C*2/*c*	Mo *K*α radiation, λ = 0.71073 Å
Hall symbol: -C 2yc	Cell parameters from 4533 reflections
*a* = 21.574 (3) Å	θ = 2.5–27.5°
*b* = 4.4055 (4) Å	µ = 11.15 mm^−^^1^
*c* = 17.9316 (19) Å	*T* = 170 K
β = 112.454 (13)°	Block, colourless
*V* = 1575.1 (3) Å^3^	0.05 × 0.04 × 0.03 mm
*Z* = 4	

### Data collection

**Table:**

Stoe IPDS-1 diffractometer	1800 independent reflections
Radiation source: fine-focus sealed tube	1355 reflections with *I* > 2σ(*I*)
graphite	*R*_int_ = 0.057
φ Scans scans	θ_max_ = 27.5°, θ_min_ = 2.5°
Absorption correction: numerical (*X-SHAPE* and *X-RED32*; Stoe & Cie, 2008)	*h* = −28→26
*T*_min_ = 0.588, *T*_max_ = 0.713	*k* = −5→4
4687 measured reflections	*l* = −23→23

### Refinement

**Table:**

Refinement on *F*^2^	Secondary atom site location: difference Fourier map
Least-squares matrix: full	Hydrogen site location: inferred from neighbouring sites
*R*[*F*^2^ > 2σ(*F*^2^)] = 0.037	H-atom parameters constrained
*wR*(*F*^2^) = 0.090	*w* = 1/[σ^2^(*F*_o_^2^) + (0.052*P*)^2^] where *P* = (*F*_o_^2^ + 2*F*_c_^2^)/3
*S* = 1.00	(Δ/σ)_max_ = 0.001
1800 reflections	Δρ_max_ = 0.90 e Å^−^^3^
79 parameters	Δρ_min_ = −0.94 e Å^−^^3^
0 restraints	Extinction correction: *SHELXL97* (Sheldrick, 2008), Fc^*^=kFc[1+0.001xFc^2^λ^3^/sin(2θ)]^-1/4^
Primary atom site location: structure-invariant direct methods	Extinction coefficient: 0.00083 (16)

### Special details

**Table:**

Geometry. All e.s.d.'s (except the e.s.d. in the dihedral angle between two l.s. planes) are estimated using the full covariance matrix. The cell e.s.d.'s are taken into account individually in the estimation of e.s.d.'s in distances, angles and torsion angles; correlations between e.s.d.'s in cell parameters are only used when they are defined by crystal symmetry. An approximate (isotropic) treatment of cell e.s.d.'s is used for estimating e.s.d.'s involving l.s. planes.
Refinement. Refinement of *F*^2^ against ALL reflections. The weighted *R*-factor *wR* and goodness of fit *S* are based on *F*^2^, conventional *R*-factors *R* are based on *F*, with *F* set to zero for negative *F*^2^. The threshold expression of *F*^2^ > σ(*F*^2^) is used only for calculating *R*-factors(gt) *etc*. and is not relevant to the choice of reflections for refinement. *R*-factors based on *F*^2^ are statistically about twice as large as those based on *F*, and *R*- factors based on ALL data will be even larger.

### Fractional atomic coordinates and isotropic or equivalent isotropic displacement parameters (Å^2^)

**Table:**

	*x*	*y*	*z*	*U*_iso_*/*U*_eq_	
Cd1	0.5000	0.53865 (16)	0.7500	0.02159 (19)	
Se1	0.39046 (3)	0.19271 (15)	0.71167 (3)	0.02260 (18)	
C1	0.3440 (3)	0.3582 (16)	0.6125 (4)	0.0257 (13)	
N1	0.3131 (3)	0.4620 (16)	0.5497 (3)	0.0320 (13)	
Se2	0.49070 (3)	0.85366 (16)	0.61994 (3)	0.02357 (19)	
C2	0.5606 (4)	0.6741 (17)	0.6015 (4)	0.0293 (15)	
N2	0.6047 (4)	0.5706 (16)	0.5900 (4)	0.0381 (15)	
K1	0.32739 (8)	0.9210 (4)	0.44900 (8)	0.0291 (3)	
O1	0.2416 (3)	0.8171 (13)	0.2915 (3)	0.0375 (12)	
H1	0.1999	0.8495	0.2786	0.056*	
H2	0.2463	0.6824	0.2602	0.056*	

### Atomic displacement parameters (Å^2^)

**Table:**

	*U*^11^	*U*^22^	*U*^33^	*U*^12^	*U*^13^	*U*^23^
Cd1	0.0190 (3)	0.0260 (4)	0.0195 (3)	0.000	0.0071 (2)	0.000
Se1	0.0206 (3)	0.0229 (3)	0.0237 (3)	−0.0014 (2)	0.0078 (2)	0.0018 (2)
C1	0.022 (3)	0.026 (4)	0.033 (3)	−0.006 (3)	0.015 (3)	−0.004 (3)
N1	0.028 (3)	0.037 (4)	0.028 (3)	−0.003 (3)	0.007 (2)	0.002 (3)
Se2	0.0244 (3)	0.0258 (4)	0.0208 (3)	0.0022 (3)	0.0089 (2)	0.0020 (2)
C2	0.038 (4)	0.029 (4)	0.022 (3)	−0.004 (3)	0.013 (3)	−0.002 (3)
N2	0.047 (4)	0.033 (4)	0.048 (3)	0.008 (3)	0.033 (3)	0.002 (3)
K1	0.0348 (8)	0.0263 (8)	0.0292 (7)	0.0001 (6)	0.0155 (6)	−0.0005 (6)
O1	0.032 (3)	0.042 (3)	0.039 (3)	−0.007 (3)	0.015 (2)	−0.008 (2)

### Geometric parameters (Å, °)

**Table:**

Cd1—Se2	2.6548 (7)	Se2—C2	1.841 (7)
Cd1—Se1	2.6720 (8)	C2—N2	1.143 (10)
Se1—C1	1.827 (7)	N2—K1^iii^	2.847 (7)
C1—N1	1.161 (9)	N2—K1^iv^	2.904 (7)
N1—K1	2.806 (6)	K1—O1	2.760 (5)
N1—K1^i^	3.076 (7)	O1—H1	0.8501
N1—K1^ii^	3.083 (7)	O1—H2	0.8500
			
Se2—Cd1—Se2^v^	116.97 (4)	N1^vi^—K1—N1^ii^	63.1 (2)
Se2—Cd1—Se1	108.03 (2)	O1—K1—Se2	152.89 (13)
Se2^v^—Cd1—Se1	106.66 (2)	N1—K1—Se2	73.29 (13)
Se2—Cd1—Se1^v^	106.66 (2)	N2^iii^—K1—Se2	74.08 (15)
Se2^v^—Cd1—Se1^v^	108.03 (2)	N2^iv^—K1—Se2	81.55 (15)
Se1—Cd1—Se1^v^	110.45 (4)	N1^vi^—K1—Se2	81.53 (12)
C1—Se1—Cd1	97.2 (2)	N1^ii^—K1—Se2	129.50 (11)
N1—C1—Se1	178.5 (6)	O1—K1—K1^vi^	99.55 (13)
C1—N1—K1	138.2 (5)	N1—K1—K1^vi^	136.11 (14)
C1—N1—K1^i^	96.6 (5)	N2^iii^—K1—K1^vi^	139.52 (14)
K1—N1—K1^i^	96.89 (17)	N2^iv^—K1—K1^vi^	39.54 (13)
C1—N1—K1^ii^	105.1 (5)	N1^vi^—K1—K1^vi^	39.22 (12)
K1—N1—K1^ii^	103.6 (2)	N1^ii^—K1—K1^vi^	80.38 (13)
K1^i^—N1—K1^ii^	116.9 (2)	Se2—K1—K1^vi^	94.61 (3)
C2—Se2—Cd1	98.1 (2)	O1—K1—K1^i^	80.45 (13)
C2—Se2—K1	118.2 (2)	N1—K1—K1^i^	43.89 (14)
Cd1—Se2—K1	119.93 (3)	N2^iii^—K1—K1^i^	40.48 (14)
N2—C2—Se2	178.1 (7)	N2^iv^—K1—K1^i^	140.46 (13)
C2—N2—K1^iii^	152.8 (6)	N1^vi^—K1—K1^i^	140.78 (12)
C2—N2—K1^iv^	105.5 (6)	N1^ii^—K1—K1^i^	99.62 (13)
K1^iii^—N2—K1^iv^	99.98 (19)	Se2—K1—K1^i^	85.39 (3)
O1—K1—N1	110.08 (18)	K1^vi^—K1—K1^i^	180.00 (9)
O1—K1—N2^iii^	80.18 (19)	O1—K1—K1^ii^	92.68 (12)
N1—K1—N2^iii^	78.66 (19)	N1—K1—K1^ii^	40.35 (13)
O1—K1—N2^iv^	94.85 (19)	N2^iii^—K1—K1^ii^	111.46 (15)
N1—K1—N2^iv^	154.22 (19)	N2^iv^—K1—K1^ii^	148.48 (14)
N2^iii^—K1—N2^iv^	99.98 (19)	N1^vi^—K1—K1^ii^	76.76 (12)
O1—K1—N1^vi^	123.42 (18)	N1^ii^—K1—K1^ii^	36.10 (12)
N1—K1—N1^vi^	96.89 (17)	Se2—K1—K1^ii^	104.44 (5)
N2^iii^—K1—N1^vi^	155.49 (19)	K1^vi^—K1—K1^ii^	108.99 (4)
N2^iv^—K1—N1^vi^	73.55 (17)	K1^i^—K1—K1^ii^	71.01 (4)
O1—K1—N1^ii^	75.99 (16)	K1—O1—H1	118.5
N1—K1—N1^ii^	76.4 (2)	K1—O1—H2	126.1
N2^iii^—K1—N1^ii^	136.7 (2)	H1—O1—H2	108.6
N2^iv^—K1—N1^ii^	117.42 (19)		

Symmetry codes: (i) *x*, *y*−1, *z*; (ii) −*x*+1/2, −*y*+3/2, −*z*+1; (iii) −*x*+1, −*y*+1, −*z*+1; (iv) −*x*+1, −*y*+2, −*z*+1; (v) −*x*+1, *y*, −*z*+3/2; (vi) *x*, *y*+1, *z*.

### Hydrogen-bond geometry (Å, °)

**Table:**

*D*—H···*A*	*D*—H	H···*A*	*D*···*A*	*D*—H···*A*
O1—H2···O1^vii^	0.85	1.92	2.760 (6)	167

Symmetry codes: (vii) −*x*+1/2, *y*−1/2, −*z*+1/2.
